# A Modified Lag Screw and Cerclage Wire Technique for the Management of Sagittal Patellar Fractures: A Case Report

**DOI:** 10.7759/cureus.104745

**Published:** 2026-03-05

**Authors:** Abdullah M AlHossan, Jamil Mahmoud, Ravikiran Vanapalli

**Affiliations:** 1 College of Medicine, Alfaisal University, Riyadh, SAU; 2 Orthopedic Surgery, King Fahad Military Medical Complex, Dhahran, SAU; 3 Orthopedic Surgery, King Saud Medical City, Riyadh, SAU; 4 Orthopedics, All India Institute of Medical Sciences (AIIMS), New Delhi, IND

**Keywords:** knee trauma, lag screw fixation, orthopedic trauma surgery, sagittal patellar fracture, tension band wiring technique

## Abstract

Sagittal patellar fractures account for a minority of patellar injuries and remain insufficiently represented in the orthopedic literature, particularly with respect to standardized operative fixation techniques. These fractures pose unique biomechanical challenges due to patellofemoral joint forces and the need to address both compressive and tensile stresses across the fracture site. We report the case of a 27-year-old man who sustained a displaced medial sagittal patellar fracture (AO/OTA 34B2.1) following a high-energy motor vehicle collision. Given the degree of articular displacement and comminution, surgical fixation was performed using a hybrid construct consisting of two 3.5 mm cannulated lag screws combined with a circumferential 1.25 mm cerclage wire. This technique provided stable interfragmentary compression while reinforcing the construct against distraction forces during knee flexion. Radiographic union was achieved by three months postoperatively, with restoration of full knee range of motion and no hardware-related complications. This case demonstrates that combining lag screw fixation with circumferential cerclage wiring offers a reproducible and biomechanically sound option for managing displaced sagittal patellar fractures, particularly in high-energy injury patterns where isolated fixation methods may be insufficient.

## Introduction

Patellar fractures present a paradox in orthopedic trauma: while representing merely 1% of skeletal injuries, they pose disproportionate challenges to functional recovery [1]. Among these, sagittal patterns, accounting for 12-17% of cases, remain conspicuously understudied [2,3]. This knowledge gap becomes particularly concerning when considering the patellofemoral joint's extraordinary biomechanical demands, withstanding forces up to 7.6 times body weight during routine activities like stair climbing [4].

The paucity of evidence for sagittal pattern management stands in stark contrast to the extensive literature on transverse fractures. A recent Swedish registry analysis of 3,194 patellar fractures revealed that 90-97% of sagittal patterns were managed non-operatively, reflecting both the typically intact extensor mechanism and the uncertainty surrounding optimal surgical techniques [2]. However, high-energy trauma often produces displacement patterns that mandate surgical intervention, particularly when articular incongruity exceeds 2 mm or displacement surpasses 4 mm [5,6].

Contemporary fixation strategies have evolved considerably, yet specific protocols for sagittal patterns remain elusive. Recent meta-analyses demonstrate superior outcomes with non-metallic fixation (15.4% complication rate) compared to traditional metallic constructs (43.8%), while cannulated screw techniques show promise in reducing hardware-related morbidity [7,8]. Biomechanical studies suggest that combining fixation principles may optimize construct stability, particularly for complex fracture patterns [8].

We present a technique that addresses these challenges, a hybrid fixation technique combining cannulated lag screws with circumferential cerclage wire. This approach demonstrates how established biomechanical principles can be adapted to address the unique demands of displaced sagittal patellar fractures.

## Case presentation

A 27-year-old previously healthy man presented in July 2025 to King Saud Medical City, a Level I trauma center, following a motor vehicle collision. As an unrestrained driver in a head-on collision with airbag deployment, he sustained multiple injuries, including rib fractures and a displaced left patellar fracture.

Initial assessment

Physical examination revealed profound left knee swelling with a palpable patellar defect. Although continuity of the extensor mechanism was preserved, the patient was unable to perform active knee extension against resistance. Neurovascular status remained intact, with palpable distal pulses and preserved sensation. Notably, the overlying skin showed no breach, eliminating concerns for open fracture management.

Imaging findings

**Figure 1:**
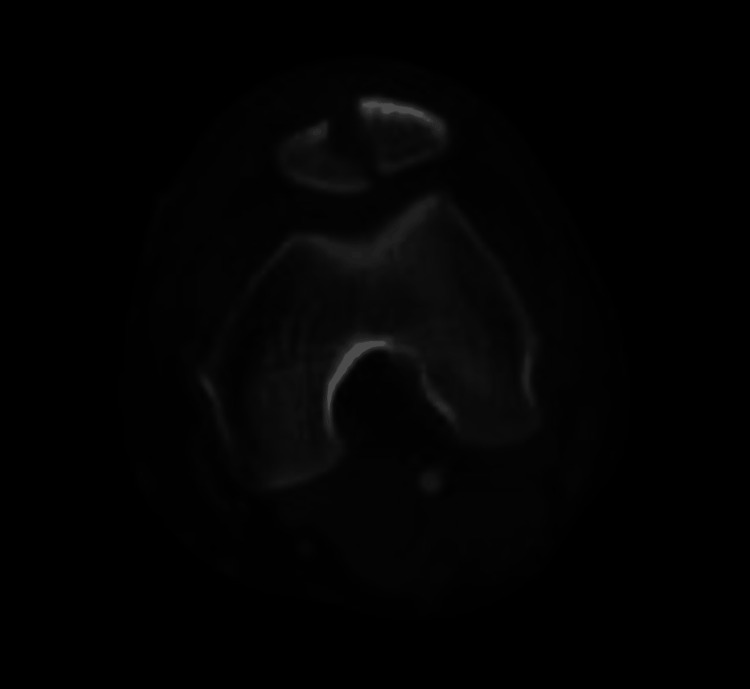
CT scan demonstrating a displaced vertical medial sagittal patellar fracture.

Radiographic evaluation demonstrated a displaced vertical medial sagittal patellar fracture (Figure 1), classified as AO/OTA 34B2.1 (partial articular, medial sagittal simple) [9]. Computed tomography (CT) refined our understanding, revealing articular displacement with focal comminution at the articular surface. No additional osteochondral fragments were identified. CT, which has been shown to alter the treatment strategy in up to 49% of complex patellar fractures, proved invaluable for surgical planning [10].

Surgical technique

Strategic Planning

Surgical intervention was deliberately delayed until hospital day 9, allowing for medical optimization and soft tissue recovery. This timing balanced the benefits of early mobilization against the risks of operating through compromised tissues. 

Surgical execution

Approach and Exposure

**Figure 2:**
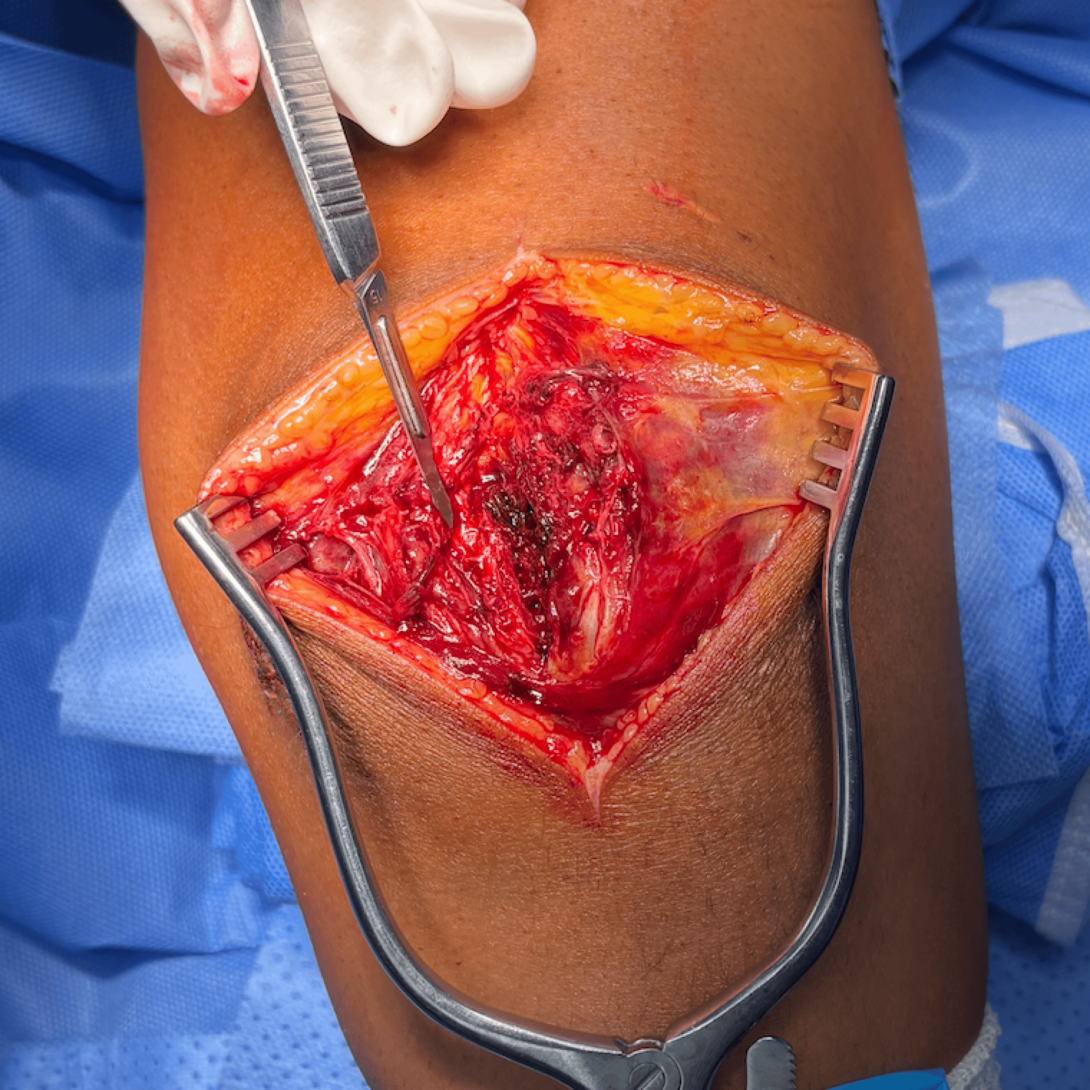
Approach to the fracture. The figure shows how the initial trauma already provided a plane through the fracture and disrupted the retinaculum, and it is not uncommon to find yourself falling inside the fracture site after opening the subcutaneous tissue.

A 10 cm direct anterior midline incision centered over the patella provided optimal exposure. Longitudinal arthrotomy through the retinaculum revealed the fracture pattern: a displaced vertical medial sagittal configuration with articular comminution but preserved tendinous attachments, as seen in Figure 2.

Reduction Technique

**Figure 3:**
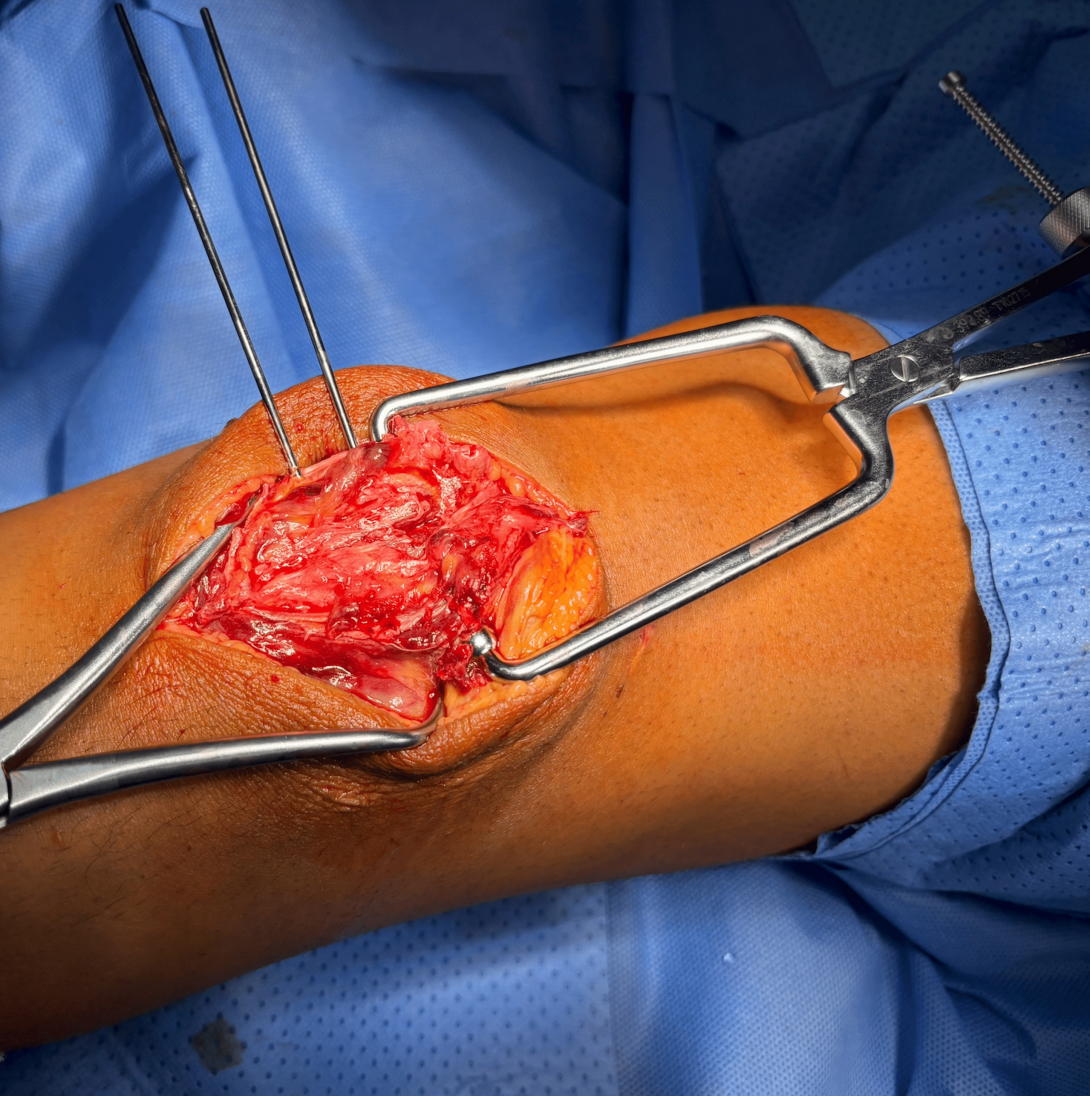
After application of the reduction clamps and two K-wires. The K-wires are 1.8 mm. They are more rigid wires than the ones used with the cannulated screws and are 1.25 mm and more malleable, which provides sufficient rigidity to maintain the reduction; in addition, the tactical feedback from inserting the wire is better with the 1.8 mm K-wire.

Pointed reduction clamps were used to facilitate anatomic reduction (Figure 3). The articular surface alignment was meticulously assessed through direct visualization, ensuring step-off elimination. Fluoroscopic confirmation in orthogonal planes verified the reduction quality before proceeding to definitive fixation. The fracture was provisionally stabilized using two 1.8 mm Kirschner wires. 

Hybrid Fixation Strategy

Phase 1 provisional stabilization: Two 1.8 mm K-wires provided temporary fixation, strategically placed perpendicular to the fracture line within the patella's anterior third. This positioning maximized cortical purchase while respecting the articular surface, a principle supported by biomechanical data favoring anterior screw placement [8].

**Figure 4:**
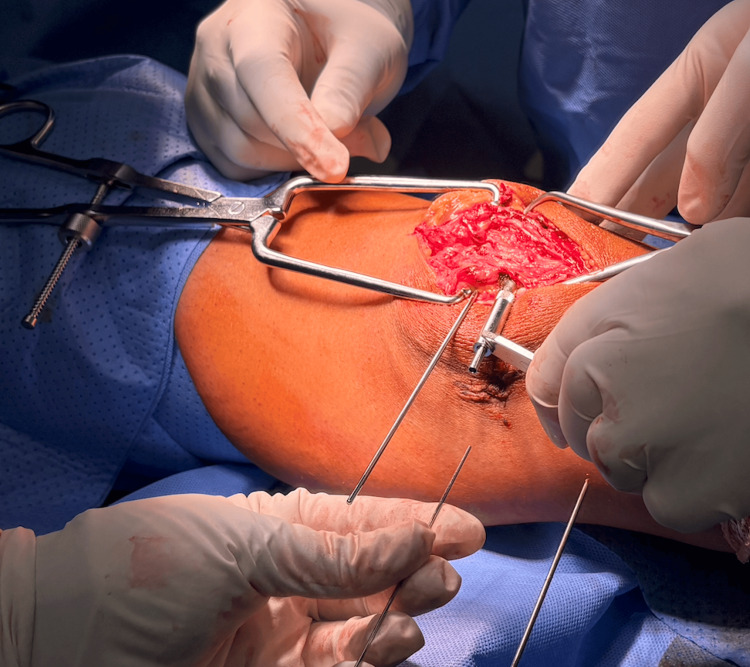
The exchange of the K-wire with the smaller guide wire The exchange of the K-wire with the smaller guide wire before inserting the screws

Phase 2 lag screw insertion: Provisional 1.8 mm wires served as initial guides. As illustrated in Figure 4, a protection sleeve was placed over each 1.8 mm wire prior to removal; this ensured that the subsequent 1.25 mm guide wires, which are compatible with the 3.5 mm cannulated screws, could be accurately inserted without losing the original trajectory. These 3.5 mm partially threaded screws (40 mm and 45 mm in length) were advanced from medial to lateral in a lag fashion to achieve stable interfragmentary compression. While recent finite element analyses advocate for full-thread screws to optimize gap reduction, we utilized partially threaded screws to prioritize maximal compression across the fracture interface [8]. 

**Figure 5:**
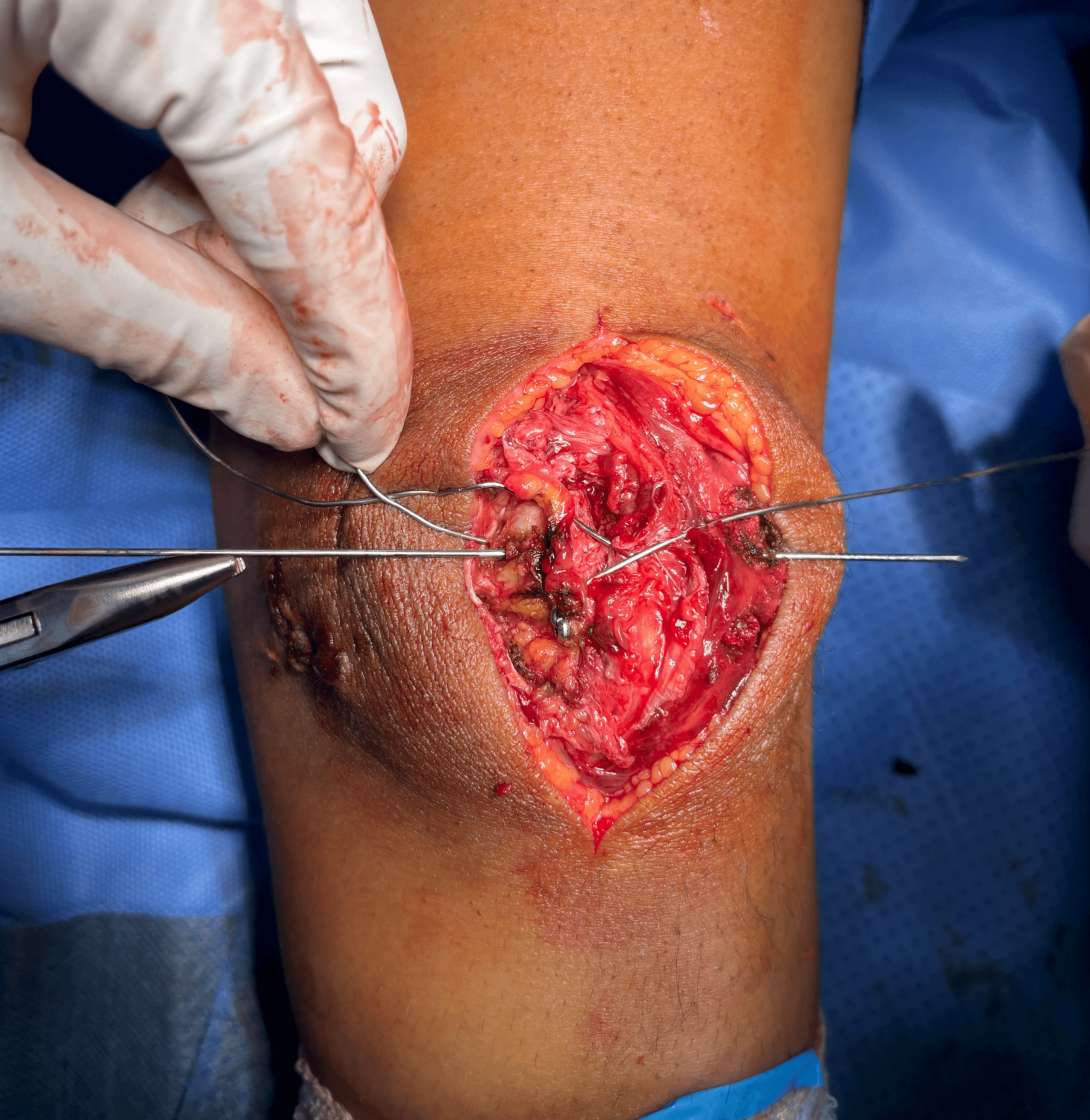
The passage of the cerclage wire. The wire is passed from medial to lateral through the cannulated screw hole and then passed again into the more superior screw medial to lateral.

Phase 3 circumferential reinforcement: A critical step of this technique involved passing a 1.25 mm stainless steel cerclage wire circumferentially around the anterior surface of the patella. This configuration created a tension band effect, converting distraction forces during knee flexion into compression at the fracture site (Figure 5).

**Figure 6:**
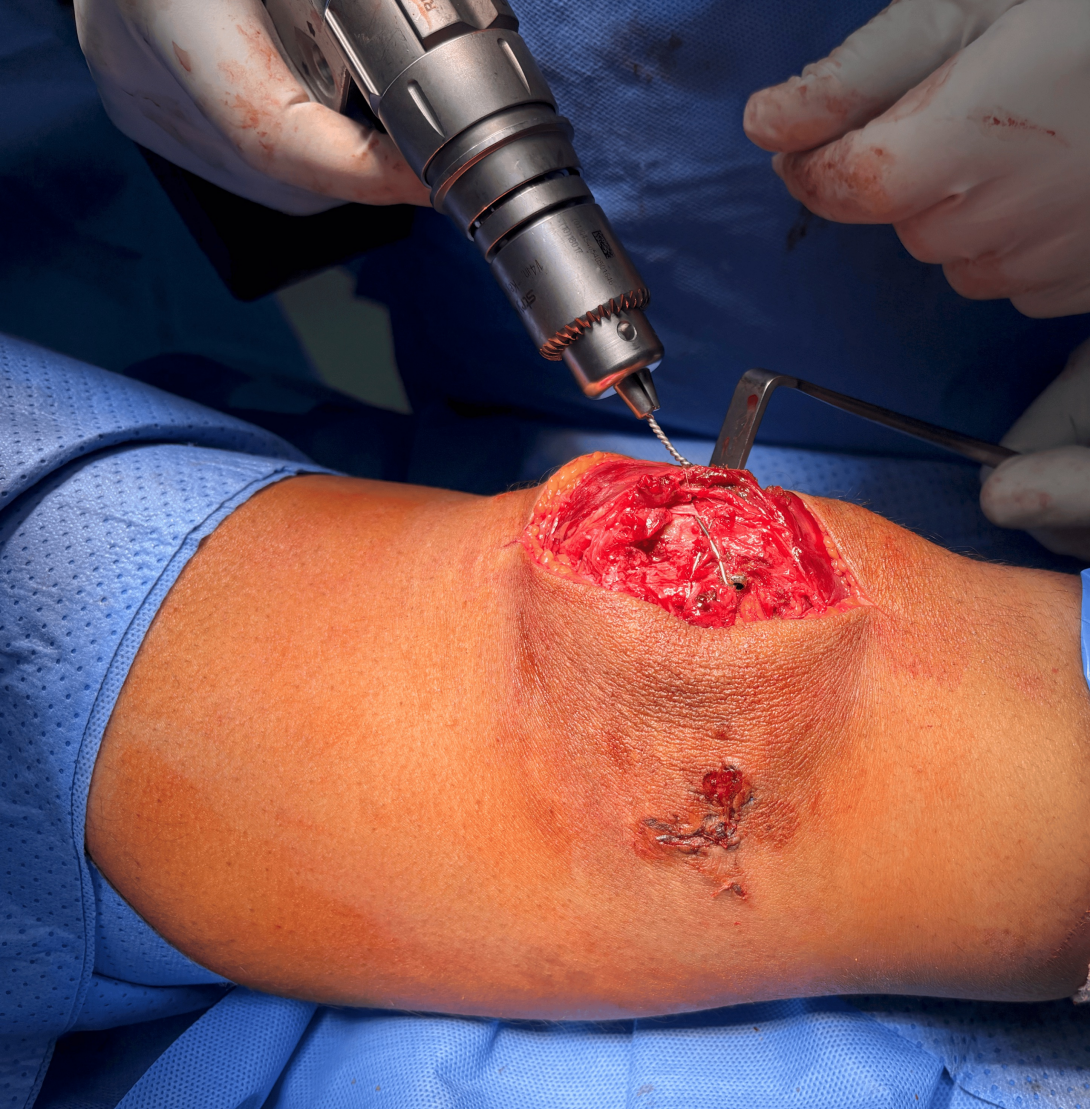
Tightening of the cerclage. Using the keyed chuck drill, we tightened both limbs of the cerclage after passing both limbs over the patella anteriorly in a figure of 8 fashion.

The cerclage wire was meticulously tensioned and secured with symmetric twisting (minimum five rotations) by placing both ends of the cerclage wires through the keyed chuck drill as shown in Figure 6. The twist was positioned away from the subcutaneous tissues to minimize postoperative irritation.

Phase 4 construct validation: Intraoperative stress testing through 0-90° range of motion confirmed construct stability. Final fluoroscopic assessment demonstrated maintained reduction, appropriate hardware positioning, and absence of articular penetration.

Postoperative protocol

Early Phase (0-2 Weeks)

Immediate postoperative care emphasized multimodal analgesia while minimizing opioid use [11]. The knee remained immobilized in extension during ambulation, though immediate static quadriceps exercises commenced to prevent muscle atrophy.

Progressive Mobilization (2-6 Weeks)

Controlled motion began at week 2, initially limited to 30° flexion and advancing 15° weekly. Weight-bearing progressed as tolerated while maintaining brace protection. This graduated approach balanced early motion benefits against fixation protection.

Advanced Rehabilitation (6-12 Weeks)

Full weight-bearing commenced at six weeks, coinciding with unrestricted range of motion exercises. Progressive resistance training restored quadriceps strength, with particular attention to eccentric control.

Return to Function (>12 Weeks)

**Figure 7:**
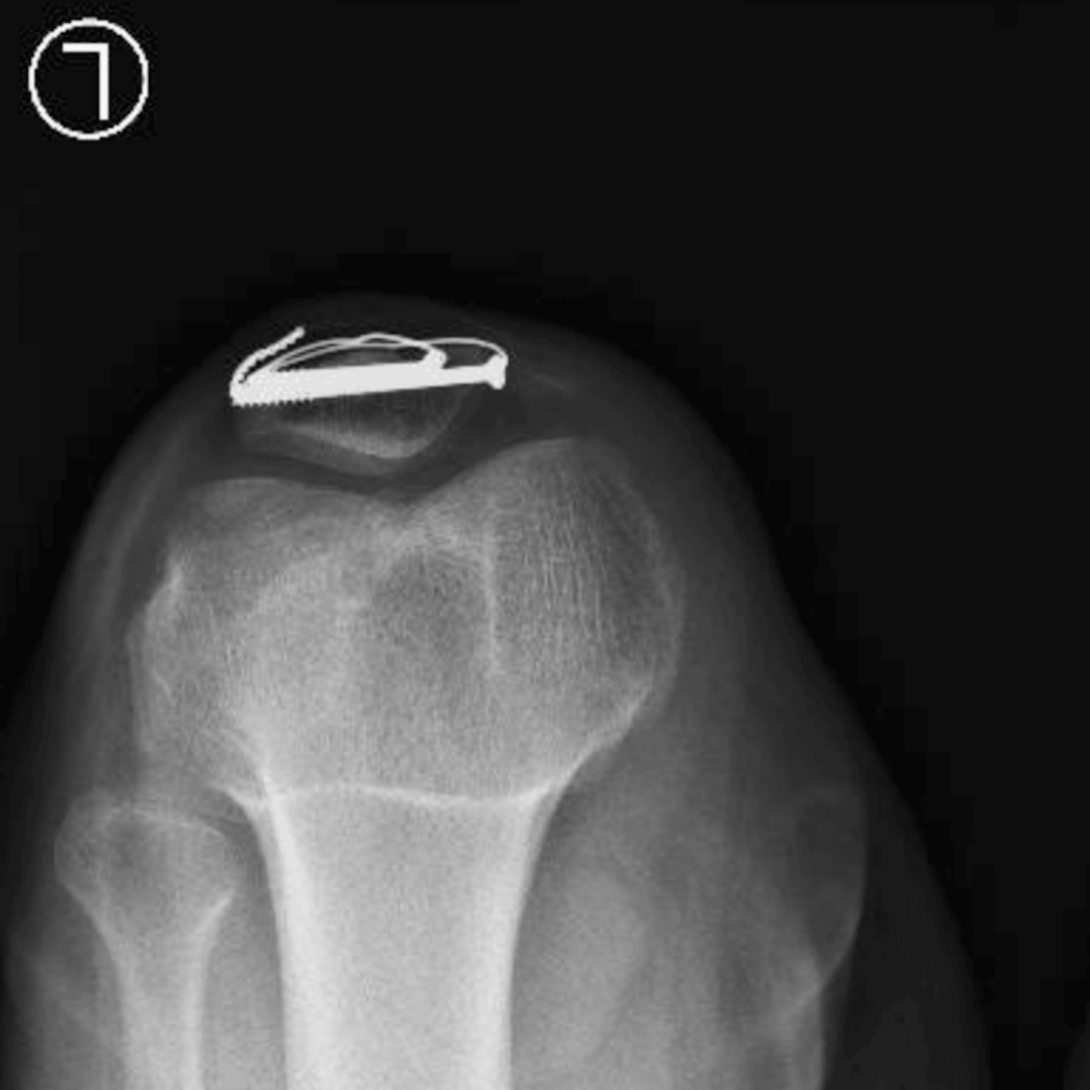
Skyline view of the left knee.

At 12 weeks mark, X-rays showed a healed fracture (Figure 7).

## Discussion

Despite significant advancements in orthopedic trauma surgery, particularly in fixation methodologies and biomechanical constructs, the surgical management of patellar fractures has demonstrated relatively limited evolution over recent decades. Current treatment presents a dilemma of achieving rigid stabilization with enhanced biomechanical strength versus minimizing hardware prominence and subsequent soft tissue irritation. This tension is reflected in contemporary treatment discussions and practical guidance, where stable fixation for early motion must be balanced against historically high rates of symptomatic hardware and reoperation after traditional metallic constructs, particularly tension band-based techniques [5,12]. Recent evidence syntheses further emphasize that implant prominence and soft tissue irritation remain key drivers of secondary procedures, motivating ongoing exploration of lower profile strategies and alternative materials where appropriate [7,12].

This case highlights a gap in current operative strategies for sagittal patellar fracture management. While sagittal patterns represent a significant minority (26%) of cases, they remain relegated to case reports and expert opinion [2,6]. Large epidemiologic studies (including registry-based work) describe overall patellar fracture burden and patterns, yet provide limited procedure-specific guidance for displaced sagittal subtypes, likely because many sagittal fractures in registry datasets are minimally displaced and therefore treated non-operatively [2,3]. In contrast, the sparse operative literature, such as small case series of rare sagittal plane injuries, often involves open fractures and/or associated intra-articular trauma, making direct extrapolation to closed displaced sagittal fractures difficult and underscoring heterogeneity in surgical decision-making [6]. Our hybrid technique directly addresses this knowledge deficit by presenting a reproducible construct and rationale tailored to the sagittal fracture plane and the clinical scenario of displacement with articular disruption.

The patellofemoral joint experiences complex loading patterns during knee motion. This demands fixation strategies that address both compressive and tensile forces. Classic biomechanical work demonstrates that quadriceps force and patellofemoral joint reaction forces rise substantially with functional activities and knee flexion, explaining why fixation must resist both separation and shear during early motion [4]. Traditional tension band wiring effectively manages transverse patterns but may inadequately compress sagittal fractures because the tension-band principle is most efficient when the fracture plane and wire orientation permit conversion of anterior tensile forces into compression at the fracture site [12]. Biomechanical and modeling studies in transverse patellar fractures show that construct behavior depends on screw type, implant placement, and the anterior band’s position, factors that can materially alter stability and gapping under load [8]. Consistent with this, biomechanical evaluation has shown that combining interfragmentary screw fixation with the tension band principle can provide improved stability over modified tension band constructs or screws alone (in transverse patterns), supporting the broader concept that “compression + tension-band reinforcement” can outperform single mechanism fixation [13]. While these data derive primarily from transverse fracture models, the underlying mechanical logic is especially relevant when sagittal patterns are exposed to different shear and rotational vectors during flexion.

Our hybrid approach combines the advantages of both techniques. The lag screws provide direct interfragmentary compression across the fracture line, supporting restoration of articular congruity. The figure-of-8 cerclage wire functions as a dynamic tension band, transforming the deforming forces of knee flexion into compressive forces at the fracture site. This construct promotes healing while permitting an early range of motion. Importantly, emerging technical descriptions of combined screw fixation with circumferential cerclage (including modern cerclage materials) reinforce the clinical appeal of combining stable compression with a low-profile reinforcing loop to support early rehabilitation while aiming to reduce symptomatic hardware [11]. In parallel, systematic review evidence suggests that non-metal fixation methods can reduce complication rates compared with traditional metallic fixation in selected contexts, highlighting a broader field-wide trend toward minimizing soft tissue irritation when biomechanical goals can still be met [7]. Although our construct uses metallic cerclage, the same principle of low-profile reinforcement with reliable compression aligns with these evolving preferences and may be adaptable to alternative materials in future comparative work.

The Swedish registry’s finding that 90-97% of sagittal patterns receive non-operative treatment likely reflects selection bias toward minimally displaced injuries [2]. Our case, with significant displacement, clearly warranted surgical intervention. Practical guidelines emphasize operative indications when extensor mechanism integrity is compromised or when displacement and articular incongruity exceed accepted thresholds, because persistent step off risks patellofemoral dysfunction and pain [5]. Additionally, fracture classification frameworks (AO/OTA) support clearer communication of pattern-specific challenges, but do not yet map cleanly to standardized fixation algorithms for displaced sagittal variants, reinforcing why technique-oriented reports remain valuable [9]. In situations where fracture morphology or comminution is uncertain on plain radiographs, advanced imaging (CT) improves characterization and can refine operative planning, especially important for uncommon patterns where implant trajectory and articular restoration are central goals [10]. Compared with reported operative sagittal plane cases in the literature that frequently involve open injuries and associated knee fractures, our closed, displaced sagittal fracture with comminution highlights a different decision context and supports the need for constructs that resist multidirectional forces during early motion [6].

The superiority of cannulated screws over K-wires for tension band constructs, demonstrated by Hoshino et al. in 109 patients, supports our technical choices [14]. Their findings of reduced soft tissue irritation and lower reoperation rates align with our favorable outcomes. These clinical results are also consistent with the broader tension-band literature, recognizing that symptomatic hardware remains a key limitation of K-wire-based constructs, often prompting removal despite union [12,14]. By using cannulated screws as the principal longitudinal elements of fixation (rather than exposed K-wires), the construct can reduce prominent metal ends and migration risk mechanisms frequently implicated in postoperative irritation while maintaining compression across the fracture plane [14]. Taken together, our fixation strategy aims to preserve the biomechanical benefits of tension-band principles while mitigating some of the most common implant-related failure modes and patient-limiting symptoms described in prior clinical series and reviews [12,14].

Limitations and future directions

Single case reports limit the generalizability of these findings. However, given the paucity of literature specifically addressing displaced sagittal patterns, detailed technical descriptions provide valuable guidance. The presence of articular comminution in our case adds complexity but demonstrates the technique's versatility.

Further biomechanical studies comparing different fixation constructs specifically for sagittal pattern patellar fractures would provide evidence-based guidance for optimal treatment selection in these challenging cases. Such studies should evaluate both mechanical properties and clinical outcomes to establish standardized treatment protocols.

## Conclusions

This case demonstrates that a hybrid construct using cannulated lag screws supplemented with a figure-of-8 cerclage can provide stable fixation for a displaced sagittal patellar fracture with articular comminution. The technique achieved restoration of articular congruity, maintained fixation through early knee motion, and resulted in successful union with functional recovery. When operative treatment is indicated for displaced sagittal patterns, combining direct interfragmentary compression with a low-profile tension-band principle may help balance mechanical stability with reduced symptomatic hardware risk.
